# Physiological consequences of rising water salinity for a declining freshwater turtle

**DOI:** 10.1093/conphys/coz054

**Published:** 2019-08-21

**Authors:** Mickey Agha, Yuzo R Yanagitsuru, Nann A Fangue, A Justin Nowakowski, Laura V Kojima, Joseph J Cech, Melissa K Riley, Janna Freeman, Dennis E Cocherell, Brian D Todd

**Affiliations:** Department of Wildlife, Fish, and Conservation Biology, University of California, Davis, One Shields Avenue, Davis, CA, USA

**Keywords:** Freshwater turtles, osmoregulation, salinity, sea-level rise

## Abstract

Sea-level rise, drought and water diversion can all lead to rapid salinization of freshwater habitats, especially in coastal areas. Increased water salinities can in turn alter the geographic distribution and ecology of freshwater species including turtles. The physiological consequences of salinization for freshwater turtles, however, are poorly known. Here, we compared the osmoregulatory response of two geographically separate populations of the freshwater Western Pond Turtle (*Actinemys marmorata*)—a species declining across its range in western North America—to three constant salinities: 0.4 ppt, 10 ppt and 15 ppt over 2 weeks. We found that turtles from a coastal estuarine marsh population regulated their plasma osmolality at lower levels than their conspecifics from an inland freshwater creek population 45 km away. Plasma osmolalities were consistently lower in estuarine marsh turtles than the freshwater creek turtles over the entire 2-week exposure to 10 ppt and 15 ppt water. Furthermore, estuarine marsh turtles maintained plasma osmolalities within 1 SD of their mean field osmolalities over the 2-week exposure, whereas freshwater creek turtles exceeded their field values within the first few days after exposure to elevated salinities. However, individuals from both populations exhibited body mass loss in 15 ppt water, with significantly greater loss in estuarine turtles. We speculate that the greater ability to osmoregulate by the estuarine marsh turtles may be explained by their reduced feeding and drinking in elevated salinities that was not exhibited by the freshwater creek population. However, due to mass loss in both populations, physiological and behavioural responses exhibited by estuarine marsh turtles may only be effective adaptations for short-term exposures to elevated salinities, such as those from tides and when traversing saline habitats, and are unlikely to be effective for long-term exposure to elevated salinity as is expected under sea-level rise.

## Teaser

Two populations of a declining western North American freshwater turtle—one coastal and one inland—were exposed to elevated water salinities of 10 ppt and 15 ppt over 2 weeks. Turtles from the coastal estuarine population had reduced feeding and drinking and maintained lower plasma osmolality levels than did those from inland.

## Introduction

Freshwater ecosystems deliver some of the most important goods and services to wildlife, fish, plants and humans around the world (Costanza *et al.*, 1997; Wilson and Carpenter, 1999). Despite their importance to ecosystem function, these systems are increasingly at risk of salinization due to land conversion, agriculture and freshwater diversion (Naiman and Turner, 2000). In the western United States, these factors are magnified by climate change, which has contributed to prolonged drought and sea-level rise (Herbert *et al.*, 2015). Drought and sea-level rise have further reduced water quality by increasing water temperatures and salinities, particularly in the Central Valley and coastal regions of California (Cardona *et al.*, 2004; Escriva-Bou *et al.*, 2017). Furthermore, past sea-level increases—20 cm in the last 100 years—coupled with future projections — 0.74 m to 1.37 m by 2100 (Ackerly *et al.*, 2018)—suggest a continuing trend of increasing mean water salinity across the San Francisco Bay Estuary (SFBE; Cayan *et al.*, 2008a, 2008b; Cloern *et al.*, 2011).

Coastal water salinities act as a limiting factor for the geographic distribution and ecology of many coastal freshwater organisms, particularly anadromous fish. For example, variable salinities pose many challenges, like increased energetic demands and physiological stress, for aquatic flora and fauna, and in some cases threaten population sustainability (Lee *et al.*, 2015; Verhille *et al.*, 2016). For reptiles and amphibians, even small increases in salinities equivalent to <1% seawater can dramatically affect physiological performance and lead to death (Hall *et al.*, 2017; Hopkins and Brodie, 2015; Findlay and Kelly, 2011). While a few freshwater reptiles have adapted to brackish water environments along coastlines, maintaining osmoregulatory homoeostasis in chronically hypertonic environments can be physiologically challenging, especially given the limited tolerance of most freshwater reptiles to salinity (Agha *et al.*, 2018; Dunson and Mazzotti, 1989).

To survive in saline waters, a few freshwater turtles are known to implement various behavioural and physiological mechanisms that allow them to temporarily occupy brackish water environments >0.5 ppt salinity for periods of hours to months, depending on the species (Agha *et al.*, 2018). These include moving between saline and freshwater habitats, reducing feeding and drinking, increasing plasma osmotic pressure relative to external environments by increasing plasma urea concentration and in some cases using a specialized lachrymal gland to excrete excess salt (Bower *et al.*, 2016; Gilles-Baillien, 1970; Harden *et al.*, 2015). While these mechanisms are effective means for tolerating saline waters for a few freshwater turtle species (Bower *et al.*, 2016; Dunson and Dunson, 1975), they are often ineffective for many others that are highly sensitive to salinities >10 ppt (Agha *et al.*, 2018). Further, in previous studies where turtles were chronically exposed to increased salinity (17.5–35 ppt), rapid body water loss occurred, which resulted in increased plasma osmolality and electrolytes, decreased muscle moisture, body mass loss and, in some cases, mortality after 7 days of salinity exposure (Bentley *et al.*, 1967; Bower *et al.*, 2016; Dunson and Seidel, 1986; Dunson, 1979; Hong *et al.*, 2014). Because these results have been found for a limited number of species, further investigation of how freshwater turtles respond to acute and chronic salinity stress is important for conservation planning, as over 90% of coastal freshwater turtles around the world will likely be affected by sea-level rise and resulting salinity by 2100 (Agha *et al.*, 2018).

The Western Pond Turtle (*Actinemys marmorata*) is the only freshwater turtle native to California (*sensu lato*) and is currently considered a Species of Special Concern due to population declines across its range in California, Oregon and Washington. Much of this decline is the result of habitat conversion, water diversion, agriculture and competition by non-native species (Jennings and Hayes, 1994; Bury and Germano, 2008; Bury *et al.*, 2012). However, mass mortalities have been associated with drought and high salinity (Leidy *et al.*, 2016; Lovich *et al.*, 2017). Although the species occurs in the estuarine reaches of the SFBE where it is exposed to variable water salinities, there is little information available on its salinity tolerance and how they may cope with salt water exposure. In addition, while salinity tolerances vary widely among species, there is little information on divergence in osmoregulatory function within wide-ranging species and among geographically separated freshwater turtle populations (Agha *et al.*, 2018). Given that Western Pond Turtles inhabit brackish waters in the SFBE and isolated freshwater areas of the Central Valley, there may be a history of evolutionary divergence and local adaptation to variable water salinities, such that differences in morphology, physiology and behaviour may be readily detected in coastal populations that have needed to compensate for past and present changes in salinity. Nonetheless, population-level differences in salt-water tolerance and associated adaptive mechanisms in response to elevated salinities are unknown for Western Pond Turtles and the physiological response to projected salinities associated with climate change and sea-level rise remains a conservation concern (Agha *et al.*, 2018). Consequently, as salinity continues to increase in concert with climate change and water management–related challenges in the Central Valley and SFBE, understanding how the widely distributed, but declining, Western Pond Turtle will respond in ecologically disparate habitats remains to be studied.

Here, we investigated the osmoregulatory responses of Western Pond Turtles from an inland freshwater creek population and a coastal estuarine marsh population to chronically elevated salinities. We compared plasma [Na^+^], [K^+^] and osmolality from individuals exposed to constant 0.4-ppt, 10-ppt and 15-ppt salinities over a 2-week period. We predicted that turtles from the coastal estuarine marsh population would be better at maintaining osmoregulatory homeostasis in the 10-ppt salinity treatment compared to their freshwater counterparts because the estuarine turtles are often exposed to elevated salinities as high as 10 ppt in the brackish water extent of their range in the SFBE. We also predicted that all turtles exposed to elevated water salinities beyond the range observed in their coastal habitats—15 ppt—would be unable to maintain osmotic and ionic homoeostasis, lose body mass due to dehydration and show corresponding increases in plasma [Na^+^], [K^+^] and osmolality that would exceed their baseline field values.

## Materials and methods

### Study locations

**Figure 1:**
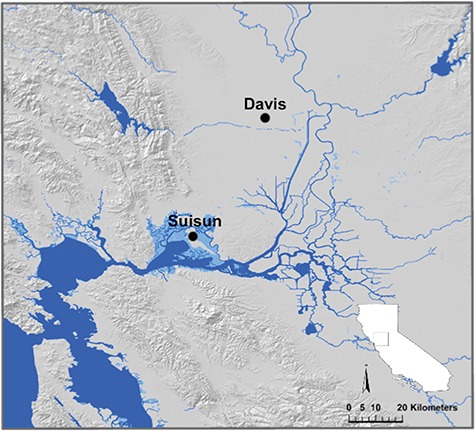
Map of western pond turtle (*A. marmorata*) collection locations including the Suisun Marsh, Solano County in the SFBE (in California) and the UC Davis Arboretum, Yolo County in the Central Valley (in California).

We collected Western Pond Turtles from two areas in California (Fig. 1): Suisun Marsh, Solano County and the University of California Davis Arboretum, Yolo County. Suisun Marsh is the largest contiguous brackish water marsh on the western coast of North America and is located at the estuarine reaches of the SFBE (Moyle *et al.*, 2014). The Suisun Marsh system includes a mixture of tidal, diked and managed marshes that are connected to a diverse array of ditches, sloughs and channels (Moyle *et al.*, 2014). On average, seasonal water salinities range from 0 ppt to 12 ppt where turtles have been observed in Suisun Marsh (Bay-Delta Live, 2018). The UC Davis Arboretum, located in the Central Valley, is a managed waterway that runs along the southern border of the campus and was formerly connected to the North Fork of Putah Creek. Located near agriculture and urban landscapes, UC Davis Arboretum is ~2.4 km in length and maintains freshwater <1-ppt salinity throughout the year (Silbernagel *et al.*, 2013). Because our two sampling sites were ecologically and environmentally disparate with regards to water salinity, we refer to Suisun Marsh turtles as ‘estuarine marsh turtles’, and UC Davis Arboretum turtles as ‘freshwater creek turtles’ below.

### Turtle capture and maintenance

Between 12 June and 28 June 2018, we collected 20 adult estuarine marsh turtles and 21 freshwater creek turtles. At both sites, we captured turtles using hoop traps baited with dead sardines. All hoop traps were ~1.8 m long, 63.5-cm hoop diameter with 3.8 cm squared mesh, made with knotted nylon netting tied to three galvanized steel hoops (Memphis Net and Twine, Memphis, TN). We determined our sample size based on variation in blood chemistry from other salinity tolerance studies on freshwater turtles (Bower *et al.*, 2016; Hong *et al.*, 2014). We individually marked captured turtles by creating notches in 1–3 marginal scutes on their carapace. For each turtle, we recorded sex, maximum straight-line carapace length (body size) using metric tree calipers and body mass with a Pesola spring scale. To ensure we were collecting adults for our study, we only retained individuals that were >120 mm maximum straight-line carapace length (Holland, 1994).

Upon capture, we transported the turtles to UC Davis where we housed them in a temperature-controlled building (21°C) in groups of two to four (populations held independently) in 1-m wide holding tanks with water temperatures maintained at 19–21°C and salinity <1 ppt. We maintained a natural photoperiod with lamps above each tank providing direct UV light. Every 2 days and after every blood draw we offered turtles canned sardines in freshwater throughout the study, and we closely monitored food consumption and removed excess food 24 h after feeding and 24 h prior to blood draw. In addition, we monitored water pH, ammonia, nitrite and nitrate daily with a colorimetric test kit (API, Calfont, PA, USA) and changed water every 1–3 days based on these water quality tests.

### Blood extraction and storage

During our study, we extracted blood in the field within 24 h of capture and at specific time points during the experiment—Day 0, 2, 4, 6, 13 and 20 (Table 1). We obtained 0.5 ml of blood from the sub-carapacial sinus of each turtle using a 23-gauge needle attached to a disposable 1-ml syringe irrigated with sodium heparin (Bower *et al.*, 2016). We recognize that sodium heparin may have affected sodium measurements; however, lithium heparin was not available during the study, and we would not expect this protocol to have any systematic effect on our comparisons across treatments or between populations. After extraction, we immediately transferred blood to a 1.5-ml polypropylene microcentrifuge tube and spun at 2700 rpm (centrifugal force ~700g) for 15 min to separate plasma. Subsequently, we transferred the plasma from each tube to sealed 1-ml cryogenic vials and immediately stored samples at −80°C. We repeated these methods to extract and store blood at pre-determined time points during our salinity tolerance experiment (Table 1).

**Table 1:** Experiment timeline for western pond turtles (*A. marmorata*) from two populations during chronic exposure to varying salinities during 2018. Pre-exposure A occurred from 12 to 28 June 2018, Pre-exposure B started on 7 July 2018 and Post-exposure release occurred from 16 to 20 August 2018.

**Experimental day**	**Event**
Pre-exposure A	All turtles captured and temporarily housed.
Pre-exposure B	All turtles separated across 12 tanks by experimental design.
0	All turtle blood draw.
2	All turtle blood draw.
3	Estuarine marsh turtles in 15-ppt treatment started to refuse food.
4	All turtle blood draw.
6	All turtle blood draw.
7	Estuarine marsh turtles in 10 ppt and 15 ppt treatments refused food.
8	Freshwater creek turtles show external signs of dehydration.
12	All turtles show external signs of dehydration.
13	All turtle blood draw.
14	10-ppt freshwater creek turtle mortality.
14	All turtles moved to freshwater.
20	All turtle blood draw.
Post-exposure	All turtles released at point of capture.

### Salinity exposure experiment

On 6 July 2018, we placed turtles in freshwater in groups of 2 to 4 across 12 independent tanks, providing all animals at least 14 days to acclimate. The 12 tanks were assigned to one of three treatments—0.4 ppt (freshwater control; henceforth FW), 10 ppt and 15 ppt—and we assigned individuals to tanks to ensure balanced sample size and distributions of sex and populations among treatments (Table 2). We selected these experimental treatments based on the upper tolerance specified for other freshwater turtle species (Bower *et al.*, 2016; Dunson and Mazzotti, 1989; Hong *et al.*, 2014) and maximum water salinities (~12 ppt) in areas where Western Pond Turtles have been observed and/or captured in Suisun Marsh (Bay-Delta Live, 2018). This salinity gradient also best represented the range of salinities that these turtles may potentially be exposed to in the near future in inland parts of their range (Palaima, 2012; Agha *et al.*, 2018). On 20 July 2018, we gradually increased water salinity to meet the assigned treatment concentration for each independent tank over a 24-h period (~3 ppt increase every 6 h) using pre-mixed Instant Ocean sea salt (Spectrum Brands, Inc, Blacksburg, VA, USA). We monitored water salinity daily throughout the experiment using a YSI 550A water quality instrument (YSI Inc, Yellow Springs, OH, USA) and adjusted as needed to maintain ±1 ppt of the specified salinity treatment, and we maintained the water level of each tank high enough to prevent complete emersion but low enough that turtles at rest could easily lift their heads to breathe. We exposed all animals to their specified treatments for 14 days and then returned them to freshwater (0.4 ppt) for 6 days.

**Table 2:** Experimental design for indoor Western Pond Turtle (*A. marmorata*) salinity tolerance study, including populations from Suisun Marsh, Solano County (estuarine marsh) and University of California Davis Arboretum, Yolo County, California (freshwater creek), treatments (Freshwater at 0.4 parts per thousand: FW, 10 parts per thousand: 10 ppt and 15 parts per thousand: 15ppt), sex (Female: F, Male: M) and each population*treatment*sex sample size. Each number in the treatment matrix (except totals) represents the number of individuals in an individual independent tank.

	**Sex**	**FW**	**10 ppt**	**15 ppt**	**Total**
**Freshwater creek**	M	4	4	4	**12**
	F	3	3	3	**9**
**Estuarine marsh**	M	4	4	4	**12**
	F	2	3	3	**8**
**Total**		**13**	**14**	**14**	**41**

We took blood samples on Days 0, 2, 4, 6 and 13 and after 6 full days in freshwater on day 20. Plasma osmolalities were analysed using a calibrated vapor pressure osmometer (Vapro® 5520, Wescor Inc., Logan, UT, USA). Plasma [Na^+^] and [K^+^] were measured using a flame photometer (Model 02655-90, Cole-Parmer Instrument Company, Vernon Hills, IL, USA).

### Statistical analysis

We compared body size of individuals from estuarine marsh and freshwater creek Western Pond Turtles using a linear model, with body size as the response variable and population and sex as fixed effects. To compare field osmolalities of estuarine marsh and freshwater creek turtles, we analysed plasma osmolality as a response variable and population, sex and body size as fixed effects using a linear model. To determine the effect of exposure to different salinities in both populations over time, we analysed response variables (plasma osmolality, [Na^+^], [K^+^] and percent body mass change) separately using linear mixed effects models (LMEs) with an interaction between time (Days 0, 2, 4, 6 and 13), population (estuarine marsh or freshwater creek), treatment (0.4 ppt, 10 ppt or 15 ppt) and sex (M or F) as fixed effects and a random intercept grouped by individual (ID) nested within Tank (~Tank|ID). Our LMEs also included an autoregressive (AR-1) correlation structure with ID nested within time as a covariate [corAR1(form ~ Time|ID)], which accounted for correlation between consecutive osmolality measurements from each individual within each population by treatment (Littell *et al.*, 2000). To assess the effect of freshwater input on Day 14 and to determine recovery in the week after salinity exposure, we reran each LME for the time period Days 13 through 20. We conducted all analyses using the software program R (R Development Core Team, 2016). We standardized explanatory variables following Cade (2015), and we determined significance of model coefficients at α = 0.05. All values are reported as mean ± standard error.

## Results

### Turtle morphology

**Figure 2:**
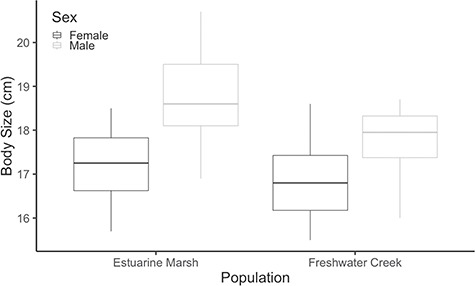
Body size (straight-line carapace length) for western pond turtles (*A. marmorata*) collected from Suisun Marsh, Solano County (estuarine marsh) and University of California Davis Arboretum, Yolo County, California (freshwater creek) populations. Box plots represent mean body size of each sex by habitat type with 25% quartiles, min and max values. Estuarine marsh turtles were larger than freshwater creek turtles (*P* = 0.01), and males were larger than females across both populations (*P* < 0.001).

Estuarine marsh turtles (18.2 ± 0.3 cm, 942 ± 35.4 g, *N* = 20) were larger on average than the freshwater creek turtles (17.3 ± 0.2 cm, 806 ± 29.7 g, *N* = 21; *P* = 0.01; Fig. 2). Across both populations, males (18.3 ± 0.2 cm, 913 ± 32.9 g, *N* = 24) were also larger than females (16.9 ± 0.2 cm, 814 ± 35.2 g, *N* = 17; *P* < 0.001; Fig. 2).

### Plasma osmolality

**Figure 3:**
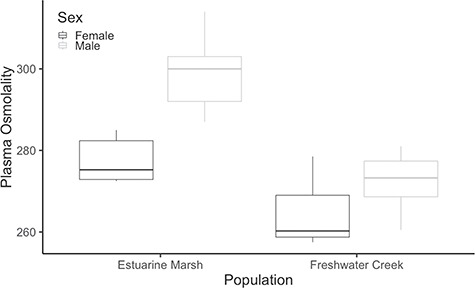
Baseline field plasma osmolality values for Western Pond Turtles (*A. marmorata*) collected from Suisun Marsh, Solano County (estuarine marsh) and University of California Davis Arboretum, Yolo County, California (freshwater creek) populations. Plasma osmolality ranges are parsed by sex and population. Box plots represent mean plasma osmolality of each sex by habitat type with 25% quartiles, min and max values. Estuarine marsh turtles had higher baseline field osmolalities when captured than did the freshwater creek turtles (*P* < 0.001), and males had higher osmolalities compared to females across both populations (*P* < 0.001).

Estuarine marsh turtles had higher baseline field osmolalities when captured than did the freshwater creek turtles (291.6 ± 2.8 versus 268.6 ± 1.7 mmol/kg, respectively; *P* < 0.001), and males had higher osmolalities compared to females (286.6 ± 2.9 versus 271.6 ± 3.1 mmol/kg, respectively; *P* < 0.001) (Fig. 3). Body size was not correlated with baseline field osmolalities (*P* = 0.19).

**Figure 4:**
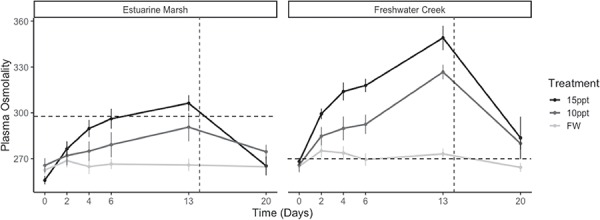
Mean plasma osmolality for Western Pond Turtles (*A. marmorata*) during chronic exposure to varying salinities (Days 0 to 20). Mean plasma osmolality values (total number of solute particles per kilogram) are parsed by population and treatment and presented with standard error (see Table 1 for sample sizes). Mean baseline field osmolality value for each population is denoted by horizontal dotted line, and when turtles were moved to freshwater is denoted by a vertical dotted line.

Overall, estuarine marsh turtles maintained lower osmolalities over time compared to their freshwater creek counterparts with salinity exposure (*P* < 0.002; Fig. 4). Over the duration of the experiment, both estuarine marsh and freshwater creek populations in FW (0.4 ppt) treatments had lower osmolalities compared to 10 (*P* < 0.001) and 15 ppt (*P* < 0.001). Estuarine marsh turtles in the 10-ppt treatment had significantly lower osmolalities compared to other population*salinity treatments (*P* = 0.01) except freshwater treatments, and freshwater creek turtles had slightly higher osmolalities compared with other population*salinity treatments (*P* = 0.06). When parsed by sex, male estuarine marsh turtles in the 10-ppt treatment had higher osmolalities compared to females in the same treatment (*P* = 0.04), and similarly, male freshwater creek turtles in the 15-ppt treatment had higher osmolalities compared to their female counterparts (*P* = 0.03).

**Figure 5:**
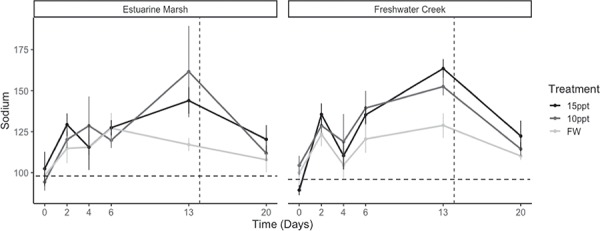
Mean [Na^+^] for Western Pond Turtles (*A. marmorata*) during chronic exposure to varying salinities (Days 0 to 20). Mean [Na^+^] (mmol/L) are parsed by population and treatment and presented with standard error (see Table 1 for sample sizes). Mean baseline field [Na^+^] value for each population is denoted by horizontal dotted line, and when turtles were moved to freshwater is denoted by a vertical dotted line.

**Figure 6:**
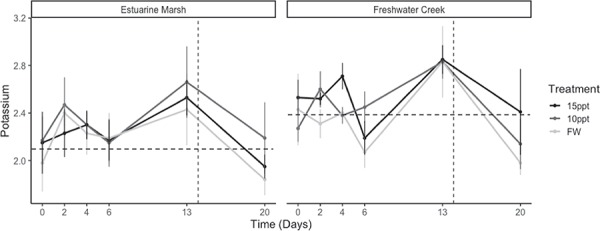
Mean [K^+^] for Western Pond Turtles (*A. marmorata*) during chronic exposure to varying salinities (Day 0 to 20). Mean [K^+^] (mmol/L) are parsed by population and treatment and presented with standard error (see Table 1 for sample sizes). Mean baseline field [K^+^] value for each population is denoted by horizontal dotted line, and when turtles were moved to freshwater is denoted by a vertical dotted line.

Estuarine marsh turtles did not differ from freshwater creek turtles based on their plasma electrolyte ([Na^+^] and [K^+^]) readings in the field ([Na^+^]: 132.15 ± 3.0 versus 132.4 ± 5.1 mmol/L, respectively, *P* = 0.85; [K^+^]: 3.1 ± 0.1 versus 2.95 ± 0.1 mmol/L, respectively, *P* = 0.63) and throughout the duration of experiment ([Na^+^]: *P* = 0.76, [K^+^]: *P* = 0.14) (Figs 5 and 6). During the exposure period, [K^+^] measurements from all individuals did not reach or exceed field [K^+^] values, whereas [Na^+^] did exceed field values from Days 6 to 13. However, [Na^+^] and [K^+^] values increased for both groups during the exposure period (*P* ≤ 0.001). Finally, we found that body size did not appear to influence plasma osmolality (*P* = 0.37) or plasma electrolytes ([Na^+^]: *P* = 0.06, [K^+^]: *P* = 0.09).

**Figure 7:**
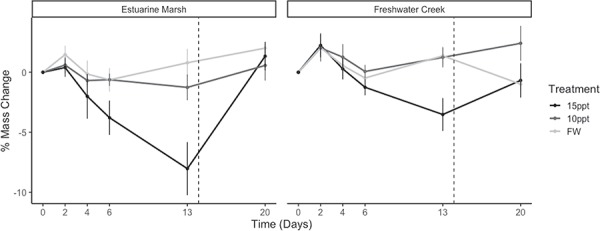
Mean percent body mass change for Western Pond Turtles (*A. marmorata*) during chronic exposure to varying salinities (Days 0 to 20). Mean percent body mass change values are parsed by population and treatment and presented with standard error (see Table 1 for sample sizes). When turtles were moved to freshwater is denoted by a vertical dotted line.

Estuarine marsh turtles had lower osmolalities than did freshwater creek turtles (*P* = 0.001) 6 days after being returned to freshwater, and in the 10- and 15-ppt treatments, they fell below field osmolality levels, whereas the freshwater creek turtles did not return to their field osmolality levels within 7 days (Fig. 7). After the return to freshwater, plasma electrolytes ([Na^+^] and [K^+^]) in both populations returned to within 1 SD of their baseline values.

### Body mass change

Estuarine marsh turtles exposed to 15 ppt had the greatest mass loss of all population*treatment groups (Fig. 5). After being returned to freshwater, estuarine marsh turtles had a slightly greater increase in body mass compared to all other population*treatment, except FW (0.4 ppt) treatments (*P* = 0.06).

### Behavioural response

Freshwater creek turtles continued to feed throughout the experiment despite showing external signs of dehydration that included sunken eyes, temporal lobes and general lethargy around Day 6. Estuarine marsh turtles in 15 ppt stopped feeding at Day 2, and estuarine marsh turtles in 10 ppt stopped feeding at Day 7. All turtles that were exposed to 15 ppt appeared lethargic and showed external signs of dehydration by Days 12 to 14. The study was halted, and all turtles were transferred to freshwater at Day 14 when a freshwater creek turtle lost its righting ability and died overnight in a 10-ppt treatment tank. After all turtles were moved to freshwater, estuarine marsh turtles in 10 ppt and 15 ppt were observed actively drinking the freshwater over multiple days and resumed eating after 6 days of freshwater exposure. Subsequently, turtles were released back to their point of capture in the wild.

## Discussion

Most of the world’s freshwater turtle species have some portion of their ranges along coastal areas and thus may have some exposure to waterways with >1 ppt salinity (Agha *et al.* 2018). Nevertheless, there has been little study of how turtle species and populations vary in their osmoregulatory ability in areas of increased salinity (but see Bower *et al.*, 2016). This represents an important knowledge gap given that projected sea-level rise and increasing diversion of freshwater to meet human demands will lead to salinization of many coastal waterways (Agha *et al.* 2018; Naiman and Turner, 2000). Here, we found that a widely distributed freshwater turtle varies in osmoregulatory strategy, with a population from an estuarine marsh maintaining lower plasma osmolality when exposed to 10- or 15-ppt salinity compared to a population from an inland freshwater creek 45 km away.

Estuarine marsh turtles maintained lower plasma osmolalities in comparison to freshwater creek turtles after exposure to elevated salinities. These disparities in plasma osmolality could be attributed to differences in feeding behaviour between the two populations, with turtles that have prior experience with occasionally salty waters restricting their drinking and feeding, responses previously seen in a few other brackish water turtle species (Bower *et al.*, 2016; Davenport and Ward, 1993). Alternatively, the discrepancy in plasma osmolality could be explained by a difference in acclimatization response between the two populations. Because plasma [Na^+^] and [K^+^] increased at similar rates in both freshwater and estuarine turtles, differences in plasma electrolytes are unlikely to explain the differences in plasma osmolality. Though we did not measure plasma urea in our study, we speculate that this interpopulation difference could be explained by increased retention of urea in the freshwater population as observed in diamond-backed terrapins (*Malaclemys terrapin*) exposed to different salinities (Gilles-Baillien, 1970). The resulting higher plasma osmolality due to increased plasma urea could maintain osmotic gradients that favor hydration even in hypertonic environments. This is in line with the lower measured mass loss in the freshwater compared to estuarine turtles. It appears then that estuarine marsh turtles osmoregulate while freshwater creek turtles osmoconform to their aquatic environment. Identifying the underlying genetic, epigenetic or physiological mechanisms that cause this difference in osmoregulatory strategy to elevated salinities between populations is an area worthy of future study.

Previous experimental studies on salinity tolerance in freshwater turtles have often gauged salinity tolerance via mass loss (Dunson and Seidel, 1986; Kinneary, 1993; Dunson and Moll, 1980). While a study has noted that turtles are able to maintain body mass under osmotically stressful conditions (Bower *et al.*, 2016), all turtles in our study lost body mass during chronic salt exposure in both salinity treatments, except for freshwater creek turtles in the 10-ppt treatments. For instance, on Day 13, estuarine marsh turtles had lost on average 1.3% of their body mass in the 10-ppt treatment tanks and 6.4% body mass in the 15-ppt treatment tanks. For freshwater creek turtles at Day 13, individuals gained on average 1.7% body mass in the 10-ppt treatment tanks and lost 3.5% in the 15-ppt treatment tanks. These mass losses corresponded with a reduction in feeding and external signs of dehydration during the experiment, with an exception for the freshwater creek turtles that continued feeding despite continuous exposure to elevated water salinities. The combination of continued feeding and increased osmolalities in freshwater creek turtles suggested that these individuals did not adaptively respond via any discernable behavioural mechanism during the study. Furthermore, individuals from both populations exhibited elevated plasma osmolalities, external signs of dehydration and high rates of body mass loss when exposed to 15-ppt brackish water for over 2 weeks. Thus, these results indicate that while estuarine marsh turtles may effectively osmoregulate under short- to medium-term periods of continuous salt exposure, long-term salinity stress (>14 days) may negatively impact both coastal and inland populations. It remains to be seen whether population-level differences in plasma osmolality are associated with underlying genetic or epigenetic differences between the populations or interpopulation differences in salinity tolerance.

Turtles from the estuarine marsh had higher baseline blood plasma osmolalities in the field compared to freshwater creek individuals, in keeping with their occasional exposure to salinities from 2–5 ppt and as great as 10 ppt in their coastal habitat. In contrast, inland waterways where the freshwater creek population resides rarely experience salinities >1 ppt. Turtles from both populations had similar plasma osmolalities after 2 weeks in freshwater before starting the experiment, suggesting that field values may change over time with freshwater input. Thus, Western Pond Turtles may facultatively osmoconform or osmoregulate, similar to Diamondback terrapins—a species restricted to brackish water environments along the Atlantic and Gulf coasts of the United States—that maintain different plasma osmolalities in waters with salinities ranging from 17.5 ppt to 34 ppt and that are able to quickly adapt to freshwater and hypo-osmotic conditions (Agha *et al.*, 2018; Gilles-Baillien, 1970). Similarly, these results correspond well with previous studies on coastal amphibians, where individuals from populations closer to the ocean were generally less reactive to salt stress than those found farther inland (Hopkins *et al.*, 2016).

Differences in body size likely represent another evolutionary adaptation to increased salinity in turtles (Agha *et al.* 2018), and we speculate that these differences may underlie some of the response variation between the two populations of Western Pond Turtles and how they responded to elevated salinities. Because rate of water loss, expressed as a proportion of total mass, generally decreases with increasing body size, it has been presumed that larger turtles have greater salinity tolerance than smaller turtles (Dunson and Heatwole, 1986; Dunson and Mazzotti, 1989; Kinneary, 1993). While we did not find an explicit effect of body size in our models, it could partly be confounded with population and sex, as estuarine marsh turtles were significantly larger than freshwater creek turtles and males were significantly larger than females. These body size differences may correspond to the observed differences between the groups—both populations and sexes—in our study. These results also correspond well with other freshwater turtle studies that have found that individuals in brackish water tend to have larger body sizes compared to their conspecifics from freshwater locales (Dunson and Mazzotti, 1989; Eisemberg *et al.*, 2015; Kinneary, 1993; Pritchard, 2001).

Western Pond Turtles benefit from the commitment of joint state-federal planning groups, private landowners and research biologists who maintain the quality and quantity of their critical freshwater habitats. However, water exports and land management in SFBE and the Central Valley create a unique predicament for fish and wildlife, most notably freshwater fauna, because they influence the balance of fresh and saline waters and water temperature (Cloern *et al.*, 2011). Water salinity is also affected by sea-level rise and lowered river inflow (Cloern *et al.*, 2011; Jeppesen *et al.*, 2015), which is currently occurring at an unprecedented rate and geographic scale (Herbert *et al.*, 2015). Furthermore, spatial and temporal variation of water salinity in coastal and inland freshwater ecosystems is exacerbated by demands for irrigation water, land clearance, industrial wastewater, river regulation, storm surges and habitat restoration (Herbert *et al.*, 2015; Jeppesen *et al.*, 2015). These natural and anthropogenic effects raise water salinities in the SFBE (Knowles, 2002; Cloern *et al.*, 2011), which can have both positive and negative effects on native flora and fauna (Moyle *et al.*, 2010). While some of these environmental impacts are known stressors for Western Pond Turtles, the effect of salinity changes across the range is yet to be addressed. Furthermore, while physiological responses observed during our study may be an inadequate mechanism for dealing with chronic exposure to elevated salinity levels, behavioural responses could lead to turtles tracking their preferred salinity levels by moving inland. However, the extent to which turtles can migrate or move inland in response to salinity changes remains unknown given the myriad habitat management strategies along coastal extents of their range combined with human development and habitat loss and fragmentation. In conclusion, our study provides resolution and necessary warning, as our data suggest that water management and climate change–mediated salinity increases in isolated or tidally influenced coastal regions may have negative consequences for the Western Pond Turtle now and in the future.
